# Pathogenicity in Chickens and Turkeys of a 2021 United States H5N1 Highly Pathogenic Avian Influenza Clade 2.3.4.4b Wild Bird Virus Compared to Two Previous H5N8 Clade 2.3.4.4 Viruses

**DOI:** 10.3390/v15112273

**Published:** 2023-11-18

**Authors:** Mary J. Pantin-Jackwood, Erica Spackman, Christina Leyson, Sungsu Youk, Scott A. Lee, Linda M. Moon, Mia K. Torchetti, Mary L. Killian, Julianna B. Lenoch, Darrell R. Kapczynski, David E. Swayne, David L. Suarez

**Affiliations:** 1 Exotic and Emerging Avian Viral Diseases Unit, Southeast Poultry Research Laboratory, U.S. National Poultry Research Center, Agricultural Research Service, U.S. Department of Agriculture, Athens, GA 30605, USA; erica.spackman@usda.gov (E.S.); lindammoon92@gmail.com (L.M.M.); david.suarez@usda.gov (D.L.S.); 2 Department of Medicine, College of Medicine, Chungbuk National University, Cheongju-si 28644, Chungbuk, Republic of Korea; 3 National Veterinary Services Laboratories, Animal and Plant Health Inspection Service, U.S. Department of Agriculture, Ames, IA 50010, USA; 4 Wildlife Services, National Wildlife Disease Program, Animal and Plant Health Inspection Service, U.S. Department of Agriculture, Fort Collins, CO 80521, USA

**Keywords:** H5N1, H5N8, highly pathogenic avian influenza virus, H5 clade 2.3.4.4, chickens, turkeys, infectivity, pathogenicity, transmission

## Abstract

Highly pathogenic avian influenza viruses (HPAIVs) of subtype H5 of the Gs/GD/96 lineage remain a major threat to poultry due to endemicity in wild birds. H5N1 HPAIVs from this lineage were detected in 2021 in the United States (U.S.) and since then have infected many wild and domestic birds. We evaluated the pathobiology of an early U.S. H5N1 HPAIV (clade 2.3.4.4b, 2021) and two H5N8 HPAIVs from previous outbreaks in the U.S. (clade 2.3.4.4c, 2014) and Europe (clade 2.3.4.4b, 2016) in chickens and turkeys. Differences in clinical signs, mean death times (MDTs), and virus transmissibility were found between chickens and turkeys. The mean bird infective dose (BID_50_) of the 2021 H5N1 virus was approximately 2.6 log_10_ 50% embryo infective dose (EID_50_) in chickens and 2.2 log_10_ EID_50_ in turkeys, and the virus transmitted to contact-exposed turkeys but not chickens. The BID_50_ for the 2016 H5N8 virus was also slightly different in chickens and turkeys (4.2 and 4.7 log_10_ EID_50_, respectively); however, the BID_50_ for the 2014 H5N8 virus was higher for chickens than turkeys (3.9 and ~0.9 log_10_ EID_50_, respectively). With all viruses, turkeys took longer to die (MDTs of 2.6–8.2 days for turkeys and 1–4 days for chickens), which increased the virus shedding period and facilitated transmission to contacts.

## 1. Introduction

Highly pathogenic (HP) avian influenza viruses (AIVs) of the H5 subtype A/Goose/Guangdong/1996 Eurasian lineage (Gs/GD) have caused multiple intercontinental outbreaks in domestic and wild bird populations through dissemination via migratory waterfowl [1]. The hemagglutinin (HA) gene from this lineage has diversified into multiple phylogenetic clades, with the clade 2.3.4.4 viruses becoming the predominant clade, particularly along the Eurasian flyways, in the last 9 years. Three major intercontinental outbreaks caused by H5 clade 2.3.4.4 HPAIVs occurred in 2014–2015, 2016–2017, and 2020–2023 [1,2,3,4,5,6]. In the first wave, H5N8 HPAIV spread from East Asia to Western Europe and North America [7]. These H5 viruses have been reclassified as clade 2.3.3.4c according to current nomenclature [8]. The second outbreak involved clade 2.3.4.4b H5N8 HPAIVs, which reassorted with low pathogenicity avian influenza viruses (LPAIVs) in Eurasian flyways generating many reassortants, including H5N5 and H5N6 HPAIVs [9,10,11,12]. The magnitude and breadth of the 2016–2017 intercontinental spread was much greater than in 2014–2015 [2]; however, viruses from the 2016–2017 outbreak were not detected in the Americas. Investigations on the epidemiology and phylodynamics of the H5 clade 2.3.4.4b HPAIVs revealed that wild birds played a major role in the spread of these viruses across various countries [2,13,14,15,16]. In the years following the second wave, continued circulation of clade 2.3.4.4b H5N8 viruses was observed in Eurasia [4,17]. Clade 2.3.4.4b H5N8 and H5N6 viruses were sporadically detected in Europe, East Asia, and Africa from 2018 to early 2020 [2,13,18,19,20]. The third intercontinental outbreak began as a surge in H5 HPAI cases in poultry and detections in wild birds in Europe and Asia in the fall of 2020 [21,22,23], spreading to Africa and the Americas, with ongoing cases being reported [5,6,24,25]. The current 2021–2023 epornitic is widespread in Europe, affecting many avian species, and sporadically mammals, and is even more extensive than the previous 2014–2015 and 2016–2017 events, with H5N1 HPAIVs being the most predominant subtype detected [5]. 

In January 2022, an H5N1 HPAIV from this lineage was reported in a wild bird sample collected in December 2021 in South Carolina, United States (U.S.) [26,27], becoming the second identified introduction of H5 clade 2.3.4.4 viruses into the U.S. via wild migratory birds. Phylogenetic analyses showed that these viruses were highly similar to H5N1 HPAIVs also detected in December 2021 in Newfoundland, Canada, which were closely related to H5N1 viruses found in Western Europe during the spring of 2021 [26]. Since then, the virus has spread through wild birds and has affected more than 58.79 million domestic birds in 47 States in the U.S. (as of 31 July 2023) [24]. The virus persisted in wild birds and poultry over the summer months, which was not observed during the 2014-15 U.S. outbreak. The virus has also genetically reassorted with North American wild bird LPAIVs [28] and has caused infections in many mammalian species [29]. 

The reason for the unprecedented spread of the recent H5N1 clade 2.3.4.4b virus is unclear. To increase our understanding of the epidemiology and pathobiology of these viruses in gallinaceous species and to improve control of H5N1 HPAI in poultry, we characterized the infectivity, transmissibility, and pathogenicity of an early U.S. H5N1 isolate (clade 2.3.4.4b) in the two most important poultry species, turkeys and chickens, and compared such results to those for two H5N8 HPAIVs from previous outbreaks (clades 2.3.4.4c and 2.3.4.4b). 

## 2. Material and Methods

### 2.1. Viruses

Three H5 clade 2.3.4.4 HPAIVs were used in this study: A/American Wigeon/South Carolina/22-000345-001/2021 (H5N1) (AMWI/SC/21) (H5 clade 2.3.4.4b), A/Tufted duck/Denmark/11740-LWPL/2016 (H5N8) (TUDU/Denmark/16) (H5 clade 2.3.4.4b), and A/Gyrfalcon/Washington/41088-6/2014 (H5N8) (GYRF/WA/14) (H5 clade 2.3.4.4c). AMWI/SC/21 was kindly provided by the National Veterinary Services Laboratories (NVSL), Animal and Plant Inspection Service (APHIS), the U.S. Department of Agriculture (USDA). This virus was collected by the Wildlife Services National Wildlife Disease Program, APHIS, USDA, on 31 December 2021, and has also been named AMWI/SC/2022 based on the date of confirmation [30]. TUDU/Denmark/16 (original source: Lars Larsen, Department of Veterinary and Animal Sciences, University of Copenhagen, Copenhagen, Denmark) and GYRF/WA/14 were obtained from the Southeast Poultry Research Laboratory (SEPRL) virus repository and were used in previous studies [31,32]. The working virus stocks were propagated and titrated via allantoic sac inoculation of 9-to-10-day-old embryonated chicken eggs (ECEs) using standard methods [33]. Brain heart infusion (BHI) broth (Becton Dickinson and Company; Sparks, MD, USA) was used to dilute the virus stocks to the appropriate dose. Experiments were performed in a biosafety level-3 enhanced (BSL-3E) facility in accordance with procedures approved by the U.S. National Poultry Research Center (USNPRC) Institutional Biosafety Committee, Agricultural Research Service (ARS), USDA.

### 2.2. Animals and Housing

Day-old turkeys (*Meleagris gallopavo*) were provided by a commercial producer and were reared at the USNPRC until three weeks of age. Four-week-old specific-pathogen-free white leghorn chickens (*Gallus gallus domesticus*) were obtained from the USNPRC in-house flocks. Birds were transferred to the animal BSL-3E facilities at the USNPRC, where each experimental group was housed in self-contained isolation units ventilated under negative pressure with inlet and outlet HEPA filtration. Chickens and turkeys had ad libitum access to food and water throughout the experiment. Housing and experimental procedures were reviewed and approved by the USNPRC Institutional Animal Care and Use Committee (IACUC). 

### 2.3. Experimental Design

A total of 80 chickens and 80 turkeys were used in this study. The experimental design was similar to previous studies [34,35,36]. Briefly, 10 birds of each species were bled prior to virus inoculation to confirm the absence of AIV antibodies enzyme-linked immunosorbent assay (ELISA, IDEXX AI MultiS-Screen ELISA Kit, Westbrook, ME, USA) according to the manufacturer’s protocol. Birds were divided into groups (Table 1), and birds in each group were inoculated intrachoanally with 100-fold incremental doses of the viruses to determine the mean 50% bird infective dose (BID_50_) of each virus for each bird species. The intended target doses were 2, 4, or 6 log_10_ 50% embryo infective dose (EID_50_) per bird administered in 0.1 mL. The actual titers of the challenge viruses were directly determined via egg titration using standard methods as described above. In addition, to evaluate the transmissibility of each virus, three naïve hatch-mate birds from the same species were added to each dose group 24 h after inoculation (contact-exposed birds). A group of eight hatch mates of the chickens and turkeys served as non-inoculated controls for each species.

Oropharyngeal (OP) and cloacal (CL) swabs were collected at 1, 2, 3, 4, 7, and 10 days-post-inoculation (dpi) from the inoculated birds, and at 1, 2, 3, 6, and 9 days post-contact (dpc) from the contact-exposed birds. Swabs were placed in 1 mL of BHI medium with penicillin (2000 units/mL), gentamicin (200 μg/mL), and amphotericin B (5 μg/mL) (Sigma-Aldrich, St. Louis, MO, USA), and stored at −80 °C until they were processed to determine virus shed titers. Two birds from the groups that received the higher doses of the viruses were euthanized and necropsied at 2 dpi. Skeletal muscle, lung, spleen, heart, and brain tissues were collected from these birds and stored at −80 °C for virus detection and quantification. A full series of tissues were additionally collected for histopathology and fixed in 10% neutral-buffered formalin. Immunohistochemistry was also conducted to assay for avian influenza virus antigen in tissues using a previously described method [37]. Identical tissue sets were collected from two non-inoculated hatch mates of each species to serve as negative controls.

All birds were observed for clinical signs and mortality from 0 to 11 dpi (direct inoculates) and 0 to 10 dpc (contacts). Birds showing severe clinical signs, including severe lethargy, prostration, neurological signs, or inability to eat or drink, were euthanized and counted as dead the next day for mean death time (MDT) calculations. At the end of the study, 11 dpi (or 10 dpc for contacts), surviving birds were bled and euthanized. 

Sera collected from all surviving birds was used to evaluate infection status by detecting AIV antibodies using the hemagglutination inhibition (HI) test and the agar gel immunodiffusion assay (AGID). Assays were performed using standard methods and homologous antigens [38,39]. The BID_50_ for each virus was calculated using the Reed–Muench method [40], using the criteria that birds were considered infected if they died or shed detectable levels of virus at any time and seroconverted at the end of the study.

### 2.4. Viral Titration in Swabs and Tissues

Swab and tissue samples were processed for quantitative real-time RT-PCR (qRT-PCR) to determine viral titer equivalents. A standard protocol that demonstrated the high correlation between qRT-PCR and infectious titers determined in ECEs [41] was used. Briefly, for OP and CL swab samples, total RNA was extracted using a MagMAX™−96 AI/NDV Viral RNA Isolation Kit^®^ (Thermo Fisher Scientific; Waltham, MA, USA) [42]. For tissues, samples were homogenized and resuspended in BHI media to a 10% (*w*/*v*) solution, and total RNA was extracted from the homogenates using Trizol LS reagent (Invitrogen/Thermo Fisher Scientific; Grand Island, NY, USA) and chloroform according to the manufacturer’s protocol. The resulting tissue RNA extracts were purified using an RNA Clean and Concentrator Kit (Zymo, Irvine, CA, USA), quantified with a NanoDrop™ 1000 Spectrophotometer (Thermo Fisher Scientific) following the manufacturer’s instructions, and accordingly diluted with Tris-EDTA buffer (10 mM Tris, 0.1 mM EDTA, pH 7.5) to obtain 50 ng/µL of total RNA. 

qRT-PCR was performed with the AgPath-ID one-step RT-PCR Kit (Ambion/Thermo Scientific; Grand Island, NY, USA) using a 7500 FAST real-time PCR system (Applied Biosystems, Foster City, CA, USA) as previously described [36]. Standard curves for viral RNA quantification were established with 10-fold dilutions of RNA extracted from the same titrated virus stocks used for inoculation. The lower limit of detection was 1.8 log_10_ EID_50_/mL for AMWI/SC/21, 1.9 log_10_ EID_50_/mL for TUDU/Denmark/16 (H5N8) HPAIV, and 1.8 log_10_ EID_50_/mL for GYRF/WA/14. 

### 2.5. Sequence Analyses

Whole-genome sequences of the three H5 HPAIVs were compared (AMWI/SC/21: GenBank accession numbers OQ789272-OQ789279; TUDU/Denmark: GenBank accession numbers MN708198-MN708205; and GYRF/WA/14: GenBank accession numbers KP307981-KP307988). For each isolate, eight segments were concatenated in order of decreasing segment length. Pairwise sequence identities among the concatenated genomes of the three virus isolates were calculated in Geneious Prime 2023.1.1 (Biomatters Ltd.; Auckland, New Zealand). Full-length open-reading frame nucleotide sequences were aligned using MAFFT [43]. Translated amino acid sequences were compared to identify amino acid differences between strains. A hemagglutinin (HA) phylogenetic tree was constructed including the three viruses used in this study. All Gs/GD lineage H5 HA sequences available as of April 2023 were downloaded from the GISAID (https://www.gisaid.org/, (accessed on 15 April 2023)) by selecting the clade 2.3.4 and 2.3.4.4. After removing duplicated sequences, a total of 5327 sequences were subjected to the phylogenetic analysis. A streamlined sequence analysis including duplicate removal, alignment, and the maximum-likelihood (ML) phylogenetic tree was carried out using RAxML by utilizing the Galaxy web platform [44,45]. The best-scoring ML tree was calculated based on 1000 bootstrapping and visualized in Geneious Prime 2023.1.1. 

## 3. Results 

### 3.1. Infectivity, Pathogenicity, and Transmission of the H5 HPAIVs in Chickens 

**Figure 1:**
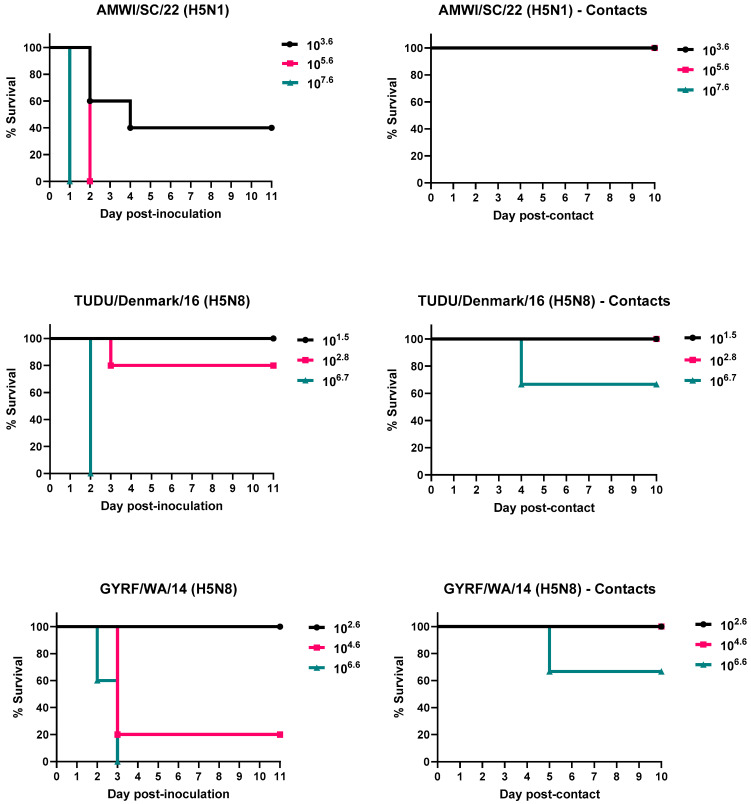
Survival curves of chickens inoculated with the H5 HPAIVs and the contact-exposed birds. Birds received low, medium, and high doses of the viruses. Contact-exposed birds were added to isolators one day post-inoculation.

**Figure 2:**
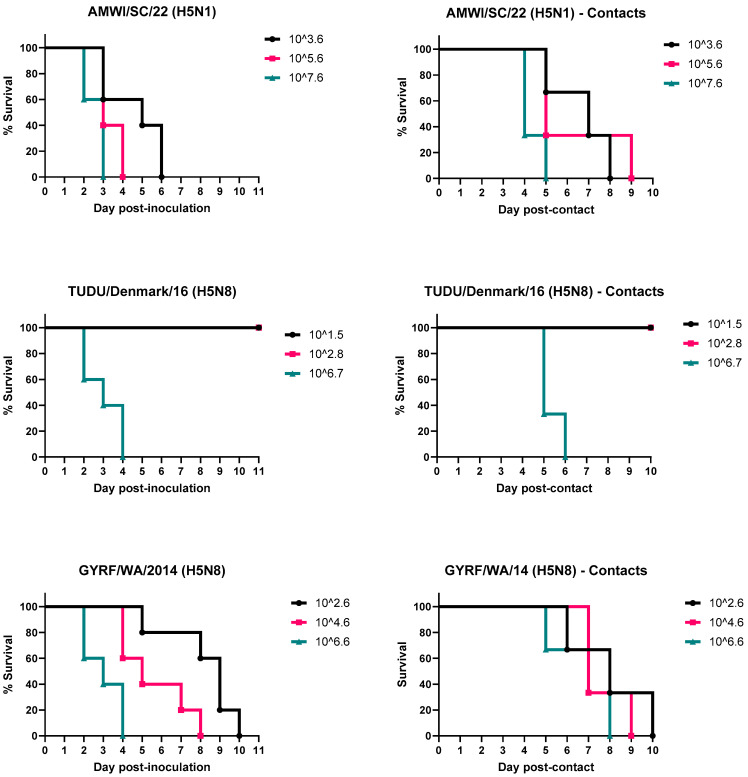
Survival curves of turkeys inoculated with the H5 HPAIVs and the contact-exposed birds. Birds received low, medium, and high doses of the viruses. Contact-exposed birds were added to isolators one day post-inoculation.

The results for virus infectivity, lethality, and transmission for chickens and turkeys are shown in Table 1 and Figure 1 and Figure 2. The titers of the inocula varied widely and were used for determining the BID_50_ for each virus.

Three out of the five chickens inoculated with the 3.6 log_10_ EID_50_ dose of AMWI/SC/21, and all chickens inoculated with the 5.6 or 7.6 log_10_ EID_50_ doses, became infected and died (MDTs between 1 and 2.7 dpi). In the two chickens in the lower-dose group that survived, the virus was detected in OP swabs at 2 dpi, but the birds were seronegative at the end of the study. With the given titers of the virus inocula, it was not possible to determine the exact 50% endpoint for BID_50_ determination. Assuming less than 50% infection at subsequent dilutions, the titer was around 2.6–3.3 log_10_ EID_50_; (depending on whether there were 0, 1, or 2 birds infected). The virus was detected in OP swabs from all contact-exposed chickens in all three dose groups, but none died or had antibodies against the virus at the end of the study. 

All five chickens inoculated with the high dose of TUDU/Denmark/16 (6.7 log_10_ EID_50_) became infected and died (MDT of 2 dpi). The virus was detected in OP swabs from all three contact-exposed birds in the high-dose group but only one died; the remaining two birds did not seroconvert. No chickens inoculated with the low dose (1.5 log_10_ EID_50_) or medium dose (2.8 log_10_ EID_50_) of this virus were infected except for one chicken in the medium-dose group, which died at 3 dpi. No contact-exposed birds in the low- or medium-dose groups were infected. The BID_50_ for TUDU/Denmark/16 in chickens was 4.2 log_10_ EID_50_. 

No chickens inoculated with the low dose of GYRF/WA/14 (2.6 log_10_ EID_50_) or the contacts in this group became infected. All chickens from the medium-dose (4.6 log_10_ EID_50_) and high-dose (6.6 log_10_ EID_50_) groups became infected, and all, except one from the medium-dose group, died (MDTs of 2.6 and 3 dpi, respectively). The surviving bird did not seroconvert. The BID_50_ for this virus was 3.9 log_10_ EID_50_. No contact-exposed birds in the medium-dose group were infected. The virus was detected in OP swabs from all three contacts from the high-dose group and from the CL route in the bird that died. The remaining two birds did not seroconvert.

No differences in clinical signs were observed in chickens infected with the different viruses or virus doses. Chickens that were severely lethargic and/or unresponsive were euthanized (83.3% of birds counted as dead). These birds also had ruffled feathers, periorbital swelling, and cyanotic combs. Some chickens died without showing clinical signs (peracute disease) (16.7%). Most of the surviving chickens did not show evidence of clinical disease. However, one and two contact-exposed chickens in the AMWI/SC/21 medium- and high-dose groups, as well as the two remaining contacts in the TD/Denmark/22 high-dose group, were less active and smaller than the rest of the birds. Non-inoculated chickens were clinically healthy throughout the experiment.

### 3.2. Infectivity, Pathogenicity, and Transmission of the H5 HPAIVs in Turkeys 

All turkeys inoculated with AMWI/SC/21, regardless of the dose given, became infected and died, with MDTs between 2.6 and 4.6 dpi depending on the virus dose; the higher the virus dose received, the shorter the MDT. The approximate BID_50_ for this virus in turkeys was 2.2 log_10_ EID_50_, assuming less than 50% survival (2/5 birds) at the next dilution; if all birds survived, the BID_50_ would be 2.6. All contact-exposed turkeys became infected and died (MDTs between 3.3 and 5.7 dpc).

No turkeys in the low- and medium-dose groups of TUDU/Denmark/16 became infected. All turkeys inoculated with the high virus dose became infected and died (MDT of 3 dpi), as well as the contact-exposed birds in this group (MDT of 4.3 dpc). The BID_50_ for this virus was 4.7 log_10_ EID_50_. 

All turkeys inoculated with GYRF/WA/14 and the contacts in these groups became infected and died. The MDT ranged from 3 to 8.2 days and correlated with dose. The MDT of the contact-exposed birds was between 5.7 and 7 dpc. The BID_50_ for this virus was approximately 0.9 log_10_ EID_50_.

Of the turkeys counted as dead, 35.7% showed no clinical signs before death. Mild lethargy was observed in the rest, which progressed to severe lethargy and prostration at which point they were euthanized (64.3% of the total dead birds). Some of the turkeys inoculated with AMWI/SC/21 also had ruffled feathers, and some inoculated with GYRF/WA/14 that were euthanized at the later timepoints had neurological signs consisting of torticollis and ataxia (5.3% and 12.5% of total dead, respectively). 

### 3.3. Viral Shedding in Chickens

**Figure 3:**
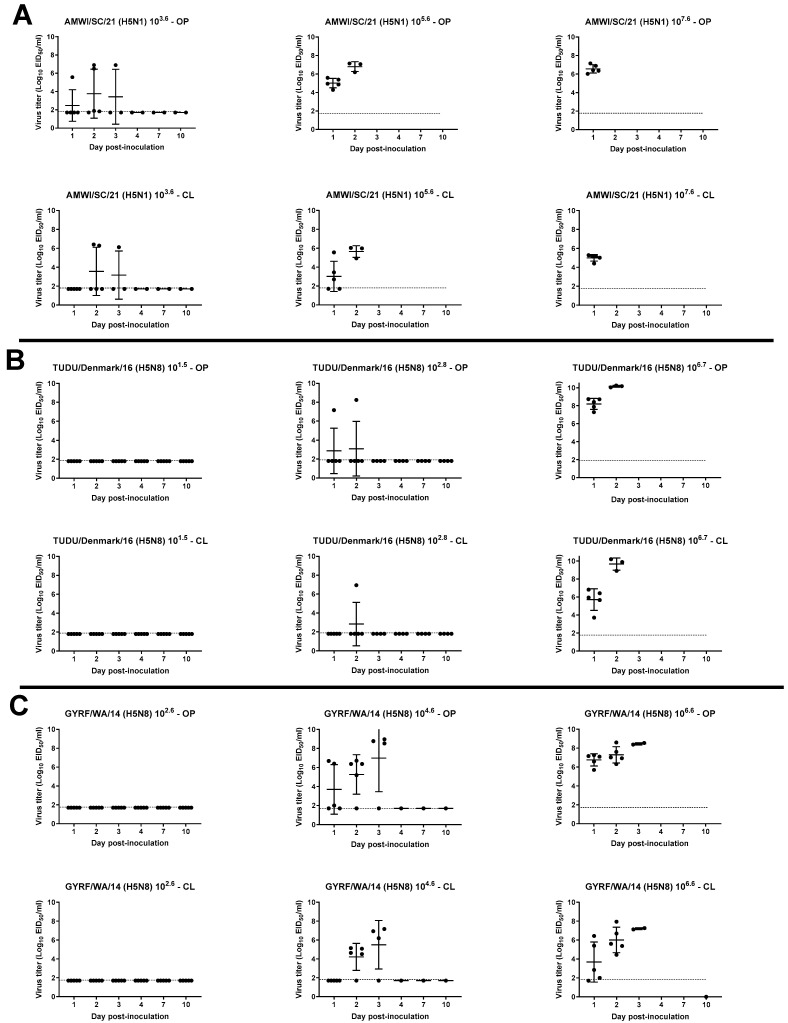
Virus shedding for chickens inoculated with different doses of the H5 HPAIVs: (**A**) AMWI/SC/21; (**B**) TUDU/Denmark/16; (**C**) GYRF/WA/14. Virus titers from oropharyngeal (OP) and cloacal (CL) swabs were determined by qRT-PCR. The dotted lines indicate the limit of detection for each virus.

**Figure 4:**
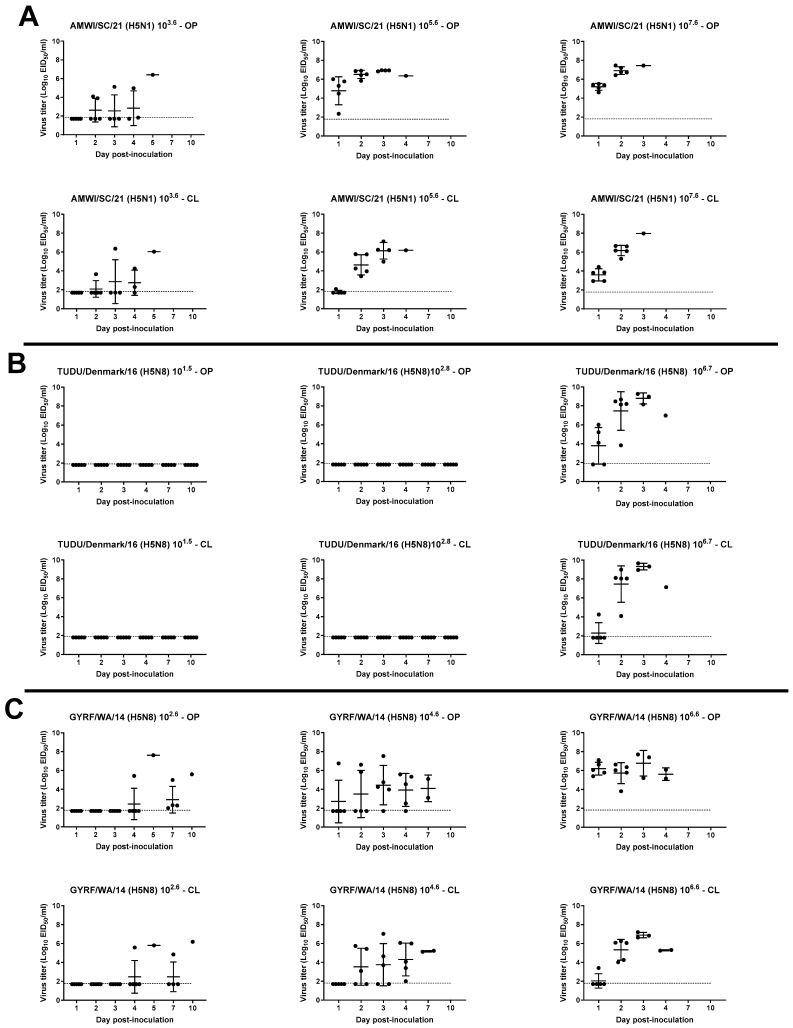
Virus shedding for turkeys inoculated with the H5 HPAIVs: (**A**) AMWI/SC/21; (**B**) TUDU/Denmark/16; (**C**) GYRF/WA/14. Virus titers from oropharyngeal (OP) and cloacal (CL) swabs were determined by qRT-PCR. The dotted lines indicate the limit of detection for each virus.

OP and CL virus shedding was examined in inoculated and contact-exposed birds by qRT-PCR (Figure 3 and Figure 4, Supplemental Figures S1 and S2). 

All chickens inoculated with the high dose of AMWI/SC/21 shed high titers of the virus by both OP and CL routes at 1 dpi (6.5 ± 0.5 and 4.9 ± 0.3 log_10_ EID_50_, respectively), and all died that day (Figure 3A). Chickens that received the medium dose also shed high OP virus titers but took a day longer to peak, after which the chickens died. Three of the five chickens that received the lower dose of the virus also shed high titers. The virus was detected in the OP swabs of one of the remaining birds in this group at 2 dpi and from all three contact-exposed chickens at 1 and 2 dpc in all groups but not in the CL swabs (Figure S1A). 

All chickens from the TUDU/Denmark/16 high-dose group and one chicken from the medium-dose group shed high titers of the virus by both OP and CL routes (8.1 ± 0.6 and 5.7 ± 1.2 log_10_ EID_50_, OP and CL, respectively, in the high-dose group at 1 dpi) (Figure 3B). Chickens that received the low dose did not have detectable levels of virus. The virus was detected in OP swabs from the three contacts in the high-dose group from 1 to 4 dpc, and one shed high virus titers by both routes and died at 4 dpc (Figure S1B). 

None of the chickens inoculated with the low dose of GYRF/WA/14 shed detectable levels of virus. High titers of the virus were shed by both OP and CL routes from all inoculated birds in the medium- and high-dose groups, with virus shedding starting earlier in the high-dose group (6.7 ± 0.6 and 3.6 ± 2.1 log_10_ EID_50_, OP and CL, respectively, 1 dpi) (Figure 3C). The virus was detected in OP swabs in two contacts from 1 to 4 dpc, and one bird shed high virus titers by both routes and died at 4 dpc (Figure S1C). 

### 3.4. Virus Shedding in Turkeys

High virus titers were detected in OP swabs starting at 1 dpi from most turkeys inoculated with the medium and high dose of AMWI/SC/21 (4.7 ± 1.4 and 5.2 ± 0.4 log_10_ EID_50_, respectively at 1 dpi) (Figure 4A). Moderate CL virus shedding was also found in the inoculated birds from the high-dose group at this timepoint (3.5 ± 0.6 log_10_ EID_50_). Between 2 and 4 dpi, high virus titers were found in CL swabs from all inoculated turkeys in these two groups, which all died by 4 dpi. Shedding in the low-dose group occurred later, and birds died between 3 and 6 dpi. All contacts shed the virus by both routes and as early as 1 dpc in the medium- and high-dose groups (Figure S2A).

Only turkeys inoculated with the high dose of TUDU/Denmark/16 shed detectable levels of virus, with high titers shed by both the OP and CL routes at 2 and 3 dpi (7.4 ± 2.0 and 7.4 ± 1.9 log_10_ EID_50_, respectively, at 2 dpi) (Figure 4B). The contacts in this group also shed the virus through both routes (Figure S2B). 

Turkeys inoculated with the high dose of GYRF/WA/14 shed high virus titers by the OP route as early as 1 dpi (6.1 ± 0.7 log_10_ EID_50_), and all birds shed by the CL route by 2 dpi (5.3 ± 1.1 log_10_ EID_50_) (Figure 4C). All birds in the groups inoculated with the low and medium doses shed the virus by both routes but reached the peak in titers at later timepoints and died later than the birds in the high-dose group. Similarly, contacts in all three groups shed the virus by both routes but at later timepoints (Figure S2C). 

### 3.5. Gross and Microscopic Lesions and Virus Detection in Tissues 

Two birds from the groups of chickens and turkeys inoculated with the high dose of each of the viruses were euthanized and necropsied at 2 dpi. Because there were no survivors in the high-dose group of chickens inoculated with AMWI/SC/21 at this timepoint, one moribund chicken from the medium-dose group was examined. Similar gross lesions were observed in all the chickens and consisted of empty intestines, multifocal necrosis in the pancreas, congested lungs, petechial hemorrhages in the thymus, cecal tonsils and skeletal muscle, and splenomegaly with pale parenchymal mottling. The gross lesions observed in the turkeys included empty intestines and dehydration. 

Microscopic lesions and viral antigen staining were similar in severity and distribution among chickens and turkeys inoculated with AMWI/SC/21 and GYRF/WA/14 and were more severe and widespread in birds inoculated with TUDU/Denmark/16. Microscopic lesions consisted of multifocal necrosis in the parenchyma of several tissues, including the brain, spleen, adrenal gland, kidney, pancreas, bursa, thymus, cecal tonsils, harderian gland, and liver, which were similar to what was reported for other H5 HPAI viruses [31,32,46,47]. Virus antigen was present in respiratory epithelial cells, and parenchymal cells of many organs, including cardiac myocytes, hepatocytes, pancreatic acinar cells, microglial cells and neurons, and kidney tubular epithelium (Table 2, Figure S3). Viral staining in vascular endothelial cells was also present in many organs from both species but was more common in chicken tissues. No gross or microscopic lesions were observed in the non-inoculated birds. No virus staining was detected in the tissues of the non-inoculated birds.

The skeletal muscle, lung, spleen, heart, and brain were also collected from the birds necropsied at 2 dpi for viral quantification via qRT-PCR (Table 3). Moderate-to-high virus titers were found in all the tissues examined regardless of the bird species or challenge virus. Titers were higher in tissues from turkeys inoculated with A/AMWI/SC/21 and TUDU/Denmark/16, compared to those from turkeys inoculated with GYRF/WA/14. Titers in tissues from chickens inoculated with TUDU/Denmark/16 were also numerically higher than what was found in tissues from birds from the other two virus groups.

### 3.6. Sequence Comparisons of the H5 HPAIVs

A phylogenetic tree of Gs/GD-lineage H5 clade 2.3.4.4 HPAIVs was constructed including the three wild bird H5 HPAIVs used in this study (Figure S4). GYRF/WA/14 clustered with clade 2.3.4.4c viruses that were introduced to the U.S. and caused the major poultry outbreak in 2014–2015. TUDU/Denmark/16 and AMWI/SC/21 belonged to a phylogenetically distinct cluster, clade 2.3.4.4b. The TUDU/Denmark/16 virus was one of the early viruses involved in the transcontinental wave of H5 clade 2.3.4.4b viruses in Europe in 2016. The clade 2.3.4.4b viruses that were persistent in wild birds in Europe eventually spread to North America, and AMWI/SC/21 was one of the earliest viruses detected in the U.S. in 2021. AMWI/SC/21 belongs to the H5N1 HPAIV genotype A1 viruses, from the initial introduction of unreassorted Eurasian-origin viruses into North America [28].

Whole-genome sequence comparison between AMWI/SC/21 and the other two viruses showed 534 amino acid differences between AMWI/SC/21 and TUDU/Denmark/16 and 805 amino acid differences between AMWI/SC/21 and GYRF/WA/14. Most of these differences were present in the Neuraminidase (NA) protein due to the different NA subtypes. The similarity between the NA from AMWI/SC/21 and TUDU/Denmark/16 was 57.02% (347 differences), and with GYRF/WA/14, the similarity was 56.32% (346 differences) (Table S1). 

Compared to the HA of AMWI/SC/21, the HA of TUDU/Denmark/16 was 97.2% similar, and the HA of GYRF/WA/14 was 92.6% similar, which could be attributed to the subclade differences, with AMWI/SC/21 and TUDU/Denmark/16 belonging to clade 2.3.4.4b and GYRF/WA/14 belonging to clade 2.3.4.4c (Figure S4). Except for PB2, the rest of the genes were over 90% similar among the three viruses.

Pairwise amino acid comparisons between AMWI/SC/21 and the other two viruses showed that AMWI/SC/21 and TUDU/Denmark/16 had a smaller number of amino acid differences than AMWI/SC/21 and GYRF/WA/14. The amino acid differences between TUDU/Denmark/16 and GYRF/WA/14 were compared in a previous study, with some residues associated with biological changes of importance [48]. Some of the changes, including PB2 R702K, HA S141P, M1 K95R, M1 K101R, M1 F144L, M1 S224N, and M1 N242K, were also found in AMWI/SC/21; some of these changes were related to increased pathogenicity in chickens [49,50,51]. Other changes, including PA T20A, PA K237E, PA R615K, and NS1 T202A, were associated with an increase in pathogenicity in ducks [52].

## 4. Discussion

Since 2014, Gs/GD lineage H5 clade 2.3.4.4 HPAIVs have rapidly emerged and spread from Asia to other parts of the world via migratory waterfowl, causing outbreaks in domestic and wild bird species [1,7,53,54]. Two intercontinental transmission events of H5 clade 2.3.4.4 viruses to North America occurred in 2014/2015 and 2021/2022. In 2014, H5N8 clade 2.3.4.4c viruses reached North America and Europe during wild bird fall migrations [7,54]. In North America, the virus reassorted with wild bird LPAIVs [55], generating an H5N2 HPAIV that subsequently spread to domestic poultry, affecting >50 million birds in the U.S. before its eradication in June 2015 [56]. Another intercontinental spread occurred in 2016/2017 involving H5 clade 2.3.4.4b viruses, which caused epizootics in wild birds and domestic poultry in Europe [57]. These viruses reassorted and generated several different NA subtypes and four groups of reassortants [4,58,59], and caused higher mortality among wild birds than the clade 2.3.4.4c viruses [12,48]. In the following years, outbreaks of H5 clade 2.3.4.4b viruses continued to occur in Europe, East Asia, and Africa, and in December 2021–January 2022, H5N1 HPAIVs were also detected in North America [26,27,60]. These viruses spread rapidly in Canada and the U.S. via wild bird movements and reassorted with wild bird LPAIVs, resulting in many genotypes [28,57], and have caused more than 325 cases in commercial flocks, 512 cases in backyard flocks, and more than 7148 detections in wild birds in the U.S. (as of 31 July 2023) [24,25]. These H5N1 viruses have achieved greater penetration into the flyways and are affecting many non-migrant wild bird species, leading to greater risks to domestic birds, especially backyard birds, than previous H5 clade 2.3.4.4 viruses. In addition, H5N1 viruses have also been detected in many mammalian species [29]. 

In the present study, we compared the infectivity, pathogenicity, and transmissibility in chickens and turkeys of one of the first 2021 H5N1 HPAIV wild bird U.S. isolates and two H5N8 HPAIV wild bird isolates from 2014 and 2016. We found that the 2021 H5N1 virus (AMWI/SC/21) was more infectious for chickens than the 2014 (GYRF/WA/14) and 2016 (TUDU/Denmark/16) H5N8 viruses. In turkeys, the 2021 H5N1 virus was more infectious than the 2016 H5N8 virus; however, the 2014 H5N8 virus was more infectious than the 2021 H5N1 virus. When comparing the results from chickens and turkeys, the approximate mean infective dose of AMWI/SC/21 was slightly lower in turkeys than chickens (~2.2 vs. ~2.6 log_10_ EID_50_), with two chickens surviving infection in the low-dose group. A difference was observed in MDTs, where chickens died 1.9, 1.4, or 1.6 days earlier than turkeys depending on the virus dose given; the higher the virus dose, the faster they died. Chickens also shed higher titers of the virus earlier than turkeys but for fewer days because of the earlier mortality. AMWI/SC/21 was detected in OP swabs of all contact-exposed chickens and turkeys, but only contact-exposed turkeys became infected and died. Contact-exposed turkeys shed viruses in moderate-to-high titers, whereas virus detection was minimal in contact-exposed chickens. As for TUDU/Denmark/16, the mean infective dose was slightly lower in chickens than in turkeys (4.2 vs. 4.7 log_10_ EID_50_), and the MDT for the high-dose groups was lower in chickens than in turkeys (2 vs. 3 dpi). The virus was detected in the OP swabs of the contact-exposed chickens in the high-dose group, but only one became infected and died; however, all contact-exposed turkeys became infected and died. Only the chicken that eventually died shed high titers by the CL route, compared to all contact-exposed turkeys that shed moderate-to-high titers by both routes. A major difference in BID_50_ was observed with GYRF/WA/14, with the virus being much more infectious for turkeys than for chickens (~0.9 vs. 3.8 log_10_ EID_50_). When comparing the MDTs in the medium- and high-dose groups, chickens died 2.6 or 0.4 days earlier, respectively, than the turkeys. The virus was detected in the OP swabs of the contact-exposed chickens in the high-dose group, but only one became infected and died; by contrast, all contact-exposed turkeys became infected and died. 

All the three H5 clade 2.3.4.4 HPAIVs used in this study caused systemic infection and high mortality in infected chickens and turkeys; however, similar to previous studies, the susceptibility to infection depended on the bird species, the virus strain, the exposure dose, and the virus’s degree of adaptation to gallinaceous species [31,32,46,47,61,62]. A recent study also examining the pathogenicity and transmission of two early U.S. H5N1 HPAIVs in chickens, including AMWI/SC/21, reported that both viruses replicated well in inoculated chickens but transmitted inefficiently to naïve contact-exposed chickens [63]. Different from our study, some contact-exposed birds did become infected with H5N1 HPAIV, but similar to our study, the virus was also transiently detected in a bird that survived. Reduced HPAIV transmission in chickens has been reported in other studies [64,65]. A study in which the pathogenicity of seven clade 2.3.4.4 viruses (two 2.3.4.4a, two 2.3.4.4b, one 2.3.4.4c, and two 2.3.4.4e) was examined in chickens showed high mortality in infected chickens but variable transmissibility; specifically, with clade 2.3.4.4b H5N8 viruses, no transmission to co-housed chickens was observed [61]. However, in another study in chickens inoculated with an H5N1 HPAIV, the rate of transmission was 100% [66]. In a follow-up study, we compared the pathobiology of AMWI/SC/21 with three other U.S. H5N1 clade 2.3.4.4b viruses resulting from the reassortment of early H5N1 HPAIVs with North American wild bird origin LPAIVs and found that all viruses had BID_50_ of <3.3 log_10_ EID_50_, and transmission to contacts was observed in the AMWI/SC/21 high-dose group (unpublished results). This variable transmissibility of HPAIVs in chickens contrasts with the 100% transmission observed in turkeys. An important caveat is that laboratory studies do not perfectly simulate transmission in the field, and housing varies, so transmissibility may not always directly compare between studies.

In this study, birds were considered infected if virus shedding and seroconversion were detected. We found that birds that shed virus through both the OP and CL routes eventually died from infection. In contrast, the virus was only detected in OP swabs for 1–2 days in chickens that survived infection. One possible explanation for this observation is that the virus detected in OP swabs was virus contracted through environmental exposure (e.g., water or feed) and may or may not be viable. Another explanation is that the virus replicated locally but did not spread systemically, and the bird’s innate immune system controlled the infection. In fact, some of the surviving chickens were smaller and less active than the non-inoculated birds, indicating possible subclinical or mild disease. The lack of detectable HI or AGID antibodies at days 10 or 11 after virus exposure suggests that the HPAIV did not replicate well, in contrast to prior studies with LPAIVs in chickens in which measurable antibodies were detected 7 days after inoculation [67]. Further studies are needed to examine the possibility of subclinical infection involving local mucosal virus replication without the systemic spread of these H5N1 HPAIVs in chickens. 

Turkeys are known to be generally more susceptible than chickens to infection with many LP and HPAIV isolates from wild waterfowl and poultry and to more effectively transmit the virus to direct contacts [36,68,69,70,71,72]. The difference in the transmissibility of Gs/GD H5 clade 2.3.4.4 HPAIVs between chickens and turkeys is also reflected in the number of field cases observed during the outbreaks in the U.S. During the H5N2 HPAI outbreak in 2015, 211 commercial poultry farms were affected, and of those, 160 were turkey operations compared to 50 chicken premises [73]. Likewise, in the current H5N1 HPAI outbreak, the number of cases in turkey farms was higher than in chicken farms: 227 turkey farms versus 61 chicken farms as of 31 July 2023 [24]. In this study, the 2014 H5N8 virus was clearly more infectious and transmissible among turkeys than among chickens. Although the infectivity of the 2016 H5N8 and 2021 H5N1 viruses was more similar between chickens and turkeys, transmission to contacts was still more efficient in turkeys.

One of the major differences in disease presentation between the chickens and turkeys in our study was the longer MDTs observed in the infected turkeys compared to chickens, which may be due to less severe vascular endothelial replication in turkeys than in chickens. Unusually long MDTs have also been reported in turkeys infected with other H5 clade 2.3.4.4 viruses [31,65], and like our study, a shorter MDT was associated with a higher infecting dose [65]. Delay in death while still shedding virus by both the OP and CL routes increases the probability of direct virus transmission or indirect transmission through cumulative environmental contamination. The high susceptibility of turkeys to HPAIV infection, coupled with high titers and duration of virus shed, would favor the spread of H5N1 HPAIVs in turkeys. Future work will continue to evaluate this lineage of virus in poultry to understand the pathobiology of evolving genotypes and how they contribute to virus spread. 

Another important difference observed in this study, apart from the earlier mortality observed in chickens compared to turkeys, was the clinical presentation of the disease. For example, all turkeys inoculated with AMWI/SC/21 became infected and died within 4.6 days regardless of dose. The chickens infected with this virus died within 2.7 days, and not all inoculated chickens died in the lower-dose group. This highlights the difference in clinical outcomes between chickens and turkeys, where chickens have a more rapid and severe response to HPAIV infection but need a higher initial dose of the virus to become infected. Blaurock et al. found that turkeys mount a different host immune response than chickens to HPAIV infection, which could explain these differences in infection outcomes [74]. It is important to note that SPF white leghorn chickens were used in this study and that results could differ in commercial layer chickens or broilers (heavy breeds) due to both rearing practices and genetics [75,76]. Also, the lesions observed in the infected birds, although characteristic of HPAIV infections, are less prominent than what has been reported in field cases of H5 clade 2.3.4.4 HPAI [77,78].

The index H5N2 and H5N8 clade 2.3.4.4c HPAIVs detected in wild birds in the U.S. in 2014 were highly adapted to waterfowl and not yet well adapted to chickens and turkeys based on the comparatively high BID_50_ of these viruses [31,46,47]. However, after multiple infection cycles in commercial flocks, the H5N2 viruses later isolated in 2015 had higher infectivity for chickens and turkeys [31,46]. The BID_50_ values for the more gallinaceous-adapted 2015 H5N2 viruses were similar to what we found with the 2021 H5N1 wild bird isolate examined in this study, indicating that this virus can more easily infect poultry than the index wild-bird 2014 H5 HPAIVs. The clade 2.3.4.4b viruses have affected many bird species in their spread through different continents. It is not clear how the virus has adapted to replicate successfully in so many different bird species, both domestic and wild, but by doing so, it has facilitated its transmission and spread.

Although TUDU/Denmark/16 and AMWI/SC/21 are more closely related, the genetic comparison of the three viruses showed many amino acid differences between them, which complicates the determination of molecular markers associated with increased infectivity and transmission in birds. However, some of the amino acid changes associated with the increased pathogenicity in chickens or ducks found between TUDU/Denmark/16 and GYRF/WA/14 [48] were also present in AMWI/SC/21. Further studies are necessary to better understand the molecular changes associated with the increased infectivity of the currently circulating H5N1 HPAIVs since they appear to be well adapted to many bird species. Molecular markers for mammalian adaptation were not found in the viruses examined in this study. These markers have rarely been identified in the clade 2.3.4.4b H5 viruses collected in birds in Europe since October 2020, suggesting that these mutations have likely emerged after transmission to mammals [5]. New isolates are continuously monitored for molecular changes that may impact infectivity and potential transmission events both in avian and mammalian species.

## 5. Conclusions

This study emphasizes the nuanced variations in pathobiology between different bird species and among different virus strains. These differences can play into the epidemiology and ecology of HPAIVs and have implications for the development of HPAIV outbreaks. The Gs/GD lineage H5 HPAIV outbreaks continue to occur, and thus, it is important to conduct pathobiology studies to inform and improve strategies to prevent and mitigate such outbreaks.

## Figures and Tables

**Table 1:** Infectivity, lethality, and transmission results from chickens and turkeys inoculated with the H5 clade 2.3.4.4 HPAIVs.

Bird Species	Virus	Dose (log_10_ EID_50_)	Inoculated					Contact-Exposed			
			# Birds Shedding Virus/Total ^1^	# Dead Birds/ Total (MDT) ^2^	# Birds HI Positive/ Total ^3^	# Birds Infected/ Total ^4^	BID_50_ (log_10_) ^5^	# Birds Shedding Virus/Total	# Dead Birds/ Total (MDT)	# Birds HI Positive/Total	# Birds Infected/ Total
Chickens	AMWI/SC/21	3.6	5/5	3/5 (2.7)	0/2	3/5	2.6 *	3/3	0/3	0/3	0/3
		5.6	5/5	5/5 (2)	na	5/5		3/3	0/3	0/3	0/3
		7.6	5/5	5/5 (1)	na	5/5		3/3	0/3	0/3	0/3
	TUDU/ Denmark/16	1.5	0/5	0/5	0/5	0/5	4.2	0/3	0/3	0/3	0/3
		2.8	1/5	1/5 (3)	0/4	1/5		0/3	0/3	0/3	0/3
		6.7	5/5	5/5 (2)	na	5/5		3/3	1/3 (4)	0/2	1/3
	GYRF/WA/14	2.6	0/5	0/5	0/5	0/5	3.9	0/3	0/3	0/3	0/3
		4.6	5/5	4/5 (3)	0/1	4/5		0/3	0/3	0/3	0/3
		6.6	5/5	5/5 (2.6)	na	5/5		3/3	1/3 (4)	0/2	1/3
Turkeys	AMWI/SC/21	3.6	4/5	5/5 (4.6)	na	5/5	2.2 *	3/3	3/3 (5.7)	na	3/3
		5.6	5/5	5/5 (3.4)	na	5/5		3/3	3/3 (5.3)	na	3/3
		7.6	5/5	5/5 (2.6)	na	5/5		3/3	3/3 (3.3)	na	3/3
	TUDU/Denmark/16	1.5	0/5	0/5	0/5	0/5	4.7	0/3	0/3	0/3	0/3
		2.8	0/5	0/5	0/5	0/5		0/3	0/3	0/3	0/3
		6.7	5/5	5/5 (3)	na	5/5		3/3	3/3 (4.3)	na	3/3
	GYRF/WA/14	2.6	5/5	5/5 (8.2)	na	5/5	0.9 *	3/3	3/3 (7)	na	3/3
		4.6	5/5	5/5 (5.6)	na	5/5		3/3	3/3 (6.7)	na	3/3
		6.6	5/5	5/5 (3)	na	5/5		3/3	3/3 (5.7)	na	3/3

na, not applicable. ^1^ #, number. ^2^ MDT, mean death time, number of dead birds × dpi/total dead birds (expressed as dpi, days post-inoculation, or dpc, days post-contact). ^3^ Mean HI titers for birds that survived (11 dpi or 10 dpc) using inoculum virus as antigen. Samples with titers <3.0 log_2_ GMT were considered negative. ^4^ Inoculated or contact-exposed birds were considered infected if they died, or shed virus and were positive for antibodies at 10 dpi (days post-inoculation) or 10 dpc (days post-contact). ^5^ BID_50_: 50% bird infective dose. * Since a lower endpoint was not achieved, 40% infectivity was assumed for the next lower dilution to calculate an approximate BID_50_.

**Table 2:** Distribution of AIV antigen in tissues collected from chickens and turkeys experimentally infected with the H5 clade 2.3.4.4. HPAIVs. Tissues were collected at 2 days post-inoculation. The scoring is expressed in the form of bird 1/bird 2.

Tissue	Chickens			Turkeys			Viral Antigen-Stained Cell Types
	AW/SC/21	TUDU/ Denmark/16	GF/WA/ 14	AW/SC/ 21	TUDU/ Denmark /16	GF/WA/ 14	
Nasal turbinate	+++	+++/+++	+++/+++	na/+++	+++/+++	+/++	Nasal epithelial cells, nasal gland epithelial cells, mononuclear cells, vascular endothelial cells
Trachea	++	+/na	+/+	++/++	++/++	+/+	Epithelial cells, mononuclear cells, vascular endothelial cells
Lung	+++	+++/+++	+++/++	++/+++	+++/++	+/++	Pneumocytes, vascular endothelial cells, mononuclear scattered in alveolar septa
Comb	+++	+++/+++	+++/+++	na/+++	na/na	na/na	Vascular endothelial cells, mononuclear cells, feather pulp
Eyelid	+++	+++/+++	+++/+++	++/na	+/na	na/na	Vascular endothelial cells, interstitium, epithelial cells
Heart	+++	+++/+++	+++/+++	++/+++	+++/++	+/na	Myocardiocytes
Brain	+++	+++/+++	+++/+++	+++/+++	+/+	+/++	Neurons, Purkinje cells, glial cells, vascular endothelial cells
Proventriculus	+	+++/+++	++/+	+/+	+/+	+/++	Epithelial cells of the mucosa and gland, mononuclear cells infiltrating mucosa and submucosa
Duodenum	-	++/++	+/-	+/+	na/+	-/+	Villi enterocytes and mononuclear cells infiltrating the mucosa and submucosa
Cecal tonsils	-/na	+++/+++	++/+	+/na	+++/++	-/+	Mononuclear cells infiltrating in mucosa and submucosa
Pancreas	+++	+++/+++	++/+	++/+++	++/++	+/+	Acinar cells, mononuclear cells, vascular endothelial cells
Liver	++	+++/++	na/++	++/++	+++/+++	-/+	Kupffer cells, hepatocytes, mononuclear cells
Kidney	++	+++/+++	++/+	+++/++	+++/+	-/na	Tubular epithelial cells, interstitial mononuclear cells, vascular endothelial cells
Adrenal gland	na	+++/+++	na/++	na/na	na/+	-/++	Corticotropic cells and infiltrating mononuclear cells
Spleen	+++	+++/+++	+++/+++	+++/+++	+++/+++	+/++	Mononuclear cells, vascular endothelial cells
Thymus	++	+++/+++	++/+	+/++	++/++	-/+	Thymic epithelium in medullar area, mononuclear cells
Cloacal bursa	+	+++/+++	+/+	+++/+	+++/na	-/+++	Mononuclear cells
Skeletal muscle	++	+/+++	-/++	++/++	+/+	-/-	Mononuclear cells
Ovaries/Testis	++	na/+++	na/na	na/+++	++/++	-/-	Tegument/interstitial tissue, endothelial cells, infiltrating mononuclear cells

na, tissue not available; (+++) widespread antigen staining; (++) common staining; (+) few stained cells; (-) no staining.

**Table 3:** Virus detection in tissues of chickens and turkeys inoculated with the H5 clade 2.3.4.4 HPAIVs. Tissues were taken from one or two birds euthanized at 2 dpi and virus titers were determined via qRT-PCR.

Bird Species	Virus	Bird ID	Muscle ^1^	Lung	Spleen	Heart	Brain
Chickens	AMWI/SC/21	CK-1	6.0	6.2	6.6	7.4	7.4
	TUDU/Denmark/16	CK-1	8.5	8.2	8.6	10.1	9.0
		CK-2	8.5	8.1	8.2	9.6	8.8
	GYRF/WA/14	CK-1	7.8	7.4	7.8	9.0	8.5
		CK-2	7.8	6.9	7.4	8.3	8.1
Turkeys	AMWI/SC/21	TK-1	6.7	6.9	7.2	7.2	7.5
		TK-2	6.3	7.8	7.5	7.7	8.0
	TUDU/Denmark/16	TK-1	6.4	7.5	8.7	7.6	6.9
		TK-2	7.0	7.8	8.9	2.9	7.4
	GYRF/WA/14	TK-1	5.0	5.3	5.0	5.7	5.2
		TK-2	6.1	5.9	5.9	6.8	6.7

^1^ Log_10_ EID_50_/g.

## Data Availability

The data that support the findings of this study are provided in the figures and tables of the article. Additional information will be available in a public archive or may be requested from the authors.

## Supplementary Materials

The following supporting information can be downloaded at: https://www.mdpi.com/article/10.3390/v15112273/s1, Figure S1. Virus shedding for contact-exposed chickens. Figure S2. Virus shedding for contact-exposed turkeys. Figure S3. Immunohistochemical staining for AIV antigen in tissues of chickens and turkeys inoculated with the H5 HPAI viruses. Figure S4. HA phylogenetic tree of Gs/GD-lineage H5 clade 2.3.4.4 HPAIVs including the three wild bird H5 HPAIVs used in this study. Table S1. Pairwise protein sequence comparison of individual genes of the H5 HPAI viruses used as challenge viruses in animal experiments.
