# Supplementary material for: Pathogenicity in Chickens and Turkeys of a 2021 United States H5N1 Highly Pathogenic Avian Influenza Clade 2.3.4.4b Wild Bird Virus Compared to Two Previous H5N8 Clade 2.3.4.4 Viruses
Source: Viruses. 2023 Nov 18;15(11):2273. doi: 10.3390/v15112273 (PMC10674317; doi:10.3390/v15112273)
Supplement: Supplementary file 1 [file viruses-15-02273-s001.zip › viruses-2701214-supplementary.pdf]

Supplementary Material

Figure S1. Virus shedding for contact chickens. A. AMWI/SC/21. B. TUDU/Denmark/16. C. GYRF/WA/14. Virus titers from oropharyngeal (OP) and cloacal (CL) swabs were determined by qRT-PCR. Dotted lines indicate the limit of detection for each virus.

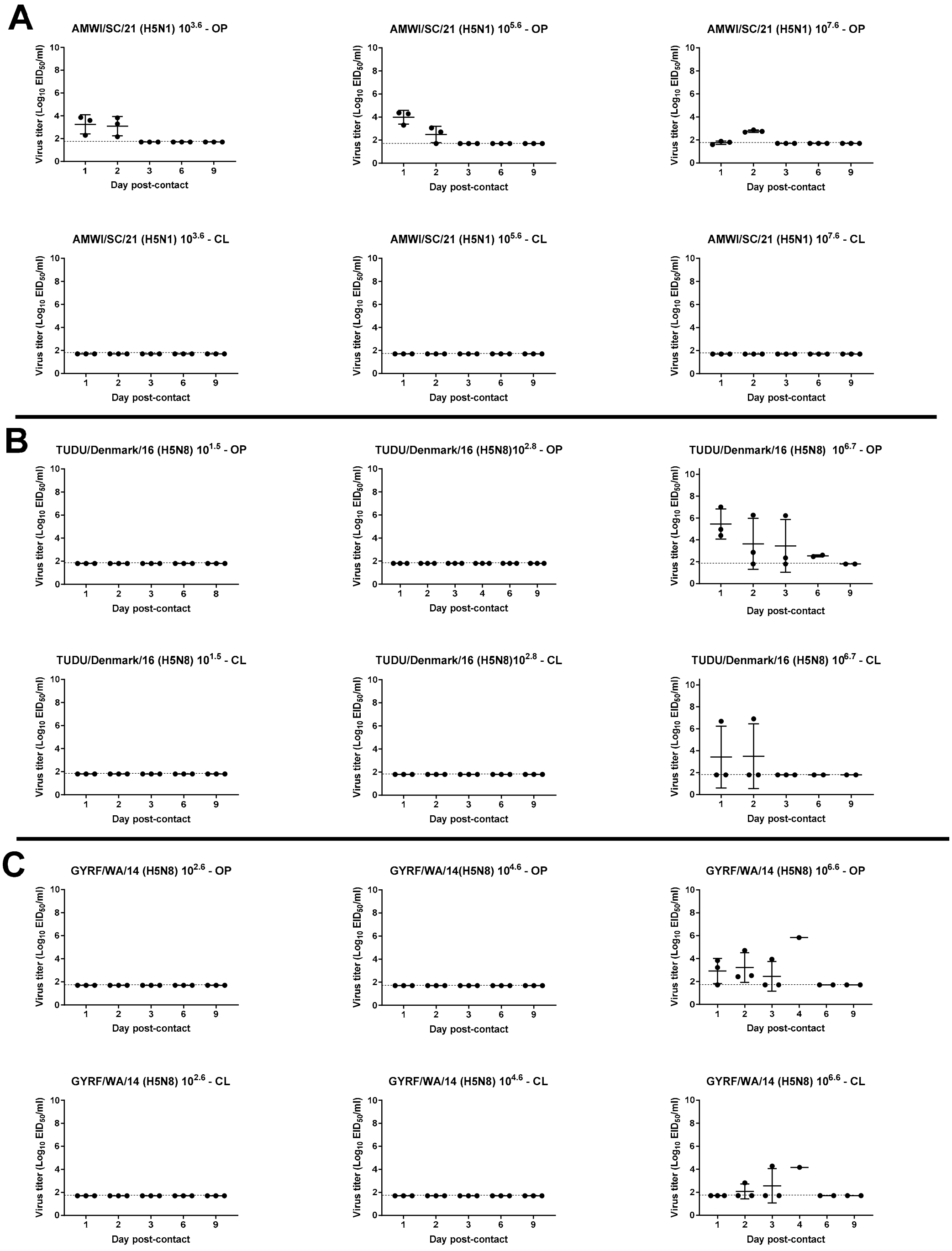

Figure S2. Virus shedding for contact turkeys. A. AMWI/SC/21. B. TUDU/Denmark/16. C. GYRF/WA/14. Virus titers from oropharyngeal (OP) and cloacal (CL) swabs were determined by qRT-PCR. Dotted lines indicate the limit of detection for each virus.

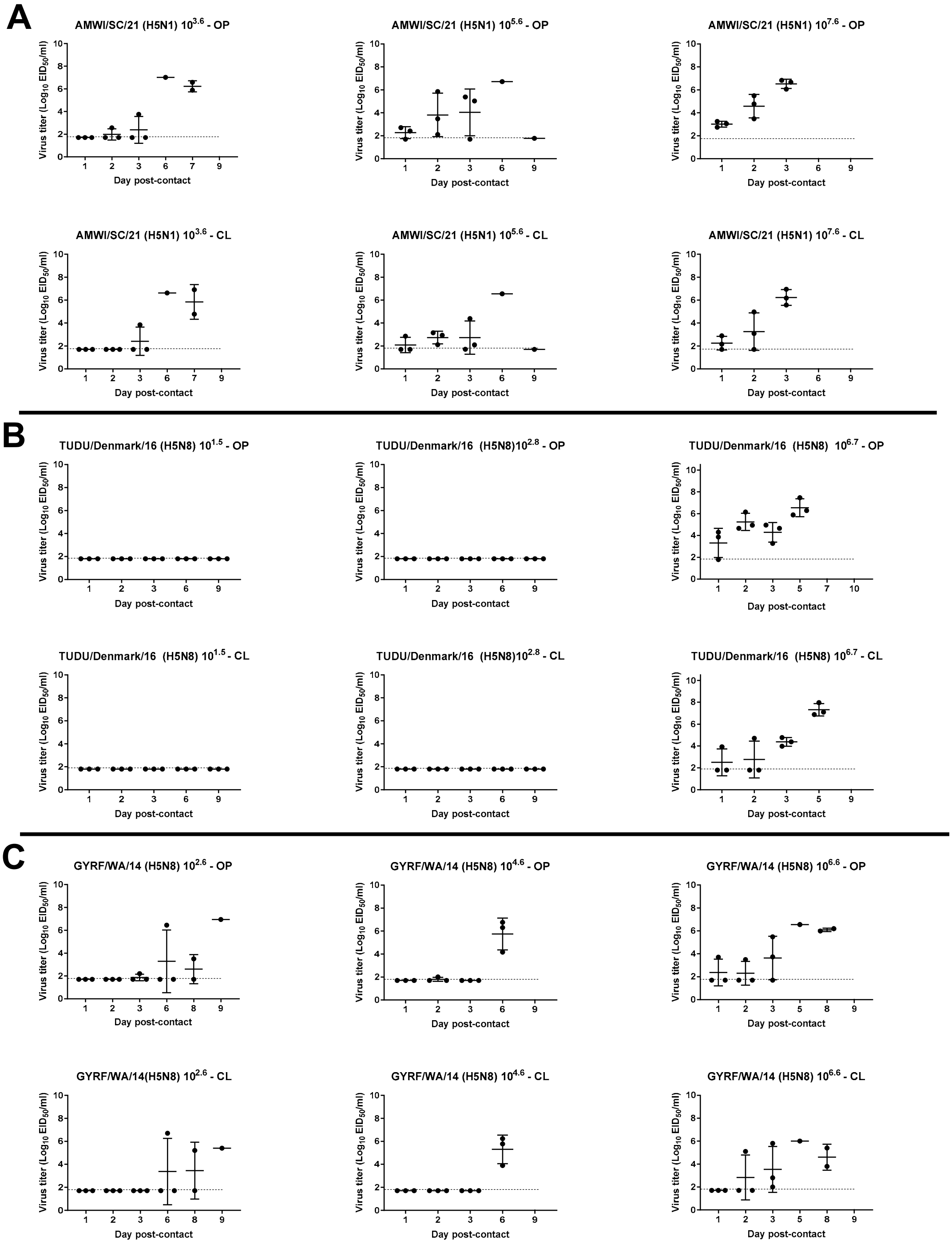

Figure S3. Immunohistochemical staining for AIV antigen in tissues of chickens and turkeys inoculated with the H5 HPAI viruses: A, C, E, I, and L, AMWI/SC/21; B, D, G, H, J, and K, TUDU/Denmark/16; F, GYRF/WA/14. Tissues collected at 2dpi. A, E, I, J, K, and L tissues from turkeys. B, C, D, F, G, and H. tissues from chickens. Virus antigens are stained red and found in the following tissues and cells: A. Nasal epithelium, vascular endothelial cells, and infiltrating mononuclear cells. B. Epithelium of air capillaries and mononuclear cells in the lung. C. Cardiac myocytes. D. Acinar cells in pancreas. E. Neurons, glial cells and vascular endothelial cells in the cerebrum. F. Vascular endothelial cells and infiltrating mononuclear cells in the eyelid submucosa. G. Tubular epithelial cells in kidney. H. Adrenal corticotropic cells. I. Mononuclear cells in the cecal tonsils. J. Mononuclear cell in the bursa. K. Mononuclear cells infiltrating the interstitial tissue in the ovary. L. Mononuclear cells in the spleen. Magnification 20X.

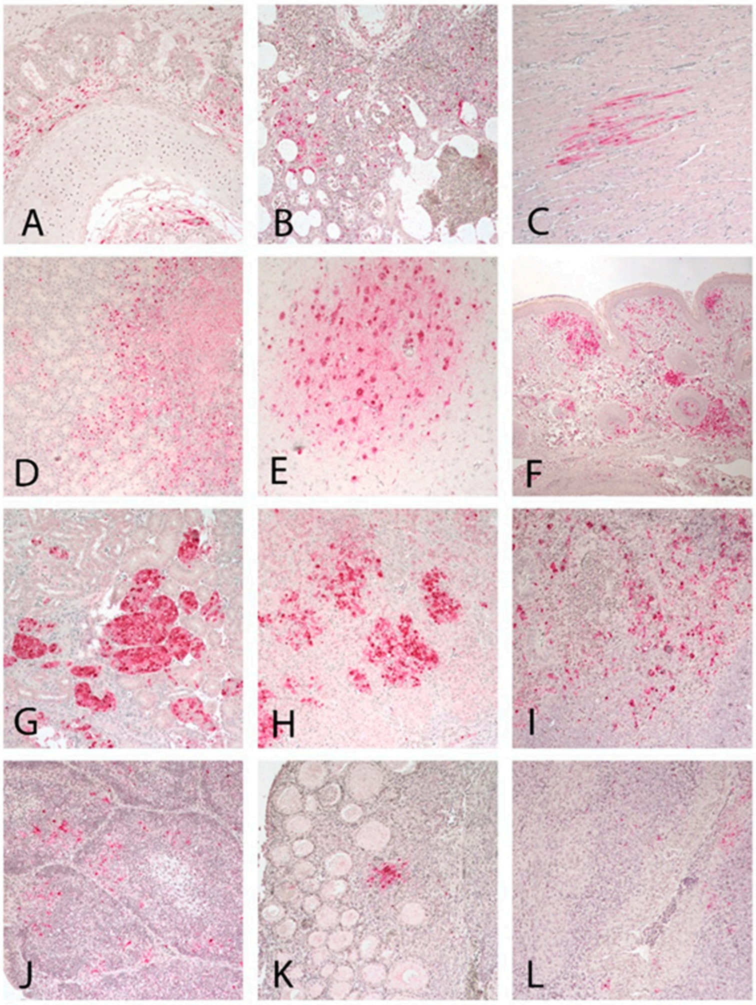

Figure S4. HA phylogenetic tree of Gs/GD-lineage H5 clade 2.3.4.4 HPAIVs including the three wild bird H5 HPAIVs used in this study. After setting an outgroup (n = 125), a deduced clade 2.3.4.4 HA tree was visualized by coloring branches of the clade 2.3.4.4; clade 2.3.4.4a, 2.3.4.4b, 2.3.4.4c, and 2.3.4.4d-h.

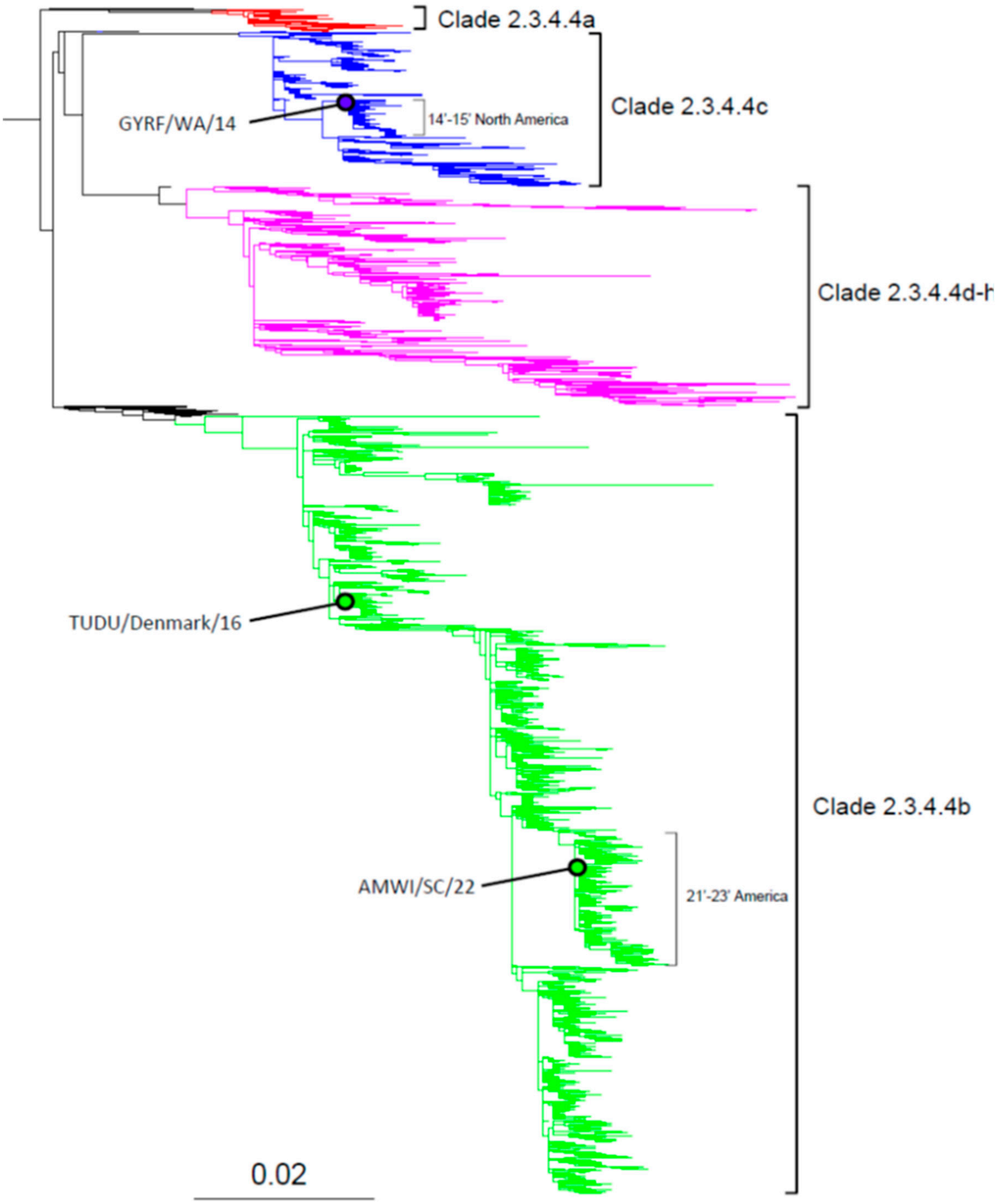

Table S1. Pairwise protein sequence comparison of individual genes of the H5 HPAI viruses used as challenge viruses in animal experiments.

	AMWI-PB2	TUDU-PB2	GYRF-PB2	AMWI-PB1	TUDU-PB1	GYRF-PB1	AMWI-PA	TUDU-PA	GYRF-PA	AMWI-NP	TUDU-NP	GYRF-NP
AMWI-PB2		90.96	86.57									
TUDU-PB2	90.96		86.66									
GYRF-PB2	86.57	86.66										
AMWI-PB1					92.43	90.28						
TUDU-PB1				92.43		90.45						
GYRF-PB1				90.28	90.45							
AMWI-PA								95.72	90.45			
TUDU-PA							95.72		91.21			
GYRF-PA							90.45	91.21				
AMWI-NP											97.32	91.51
TUDU-NP										97.32		92.38
GYRF-NP										91.51	92.38	

	AMWI-HA	TUDU-HA	GYRF-HA	AMWI-NA	TUDU-NA	GYRF-NA	AMWI-M	TUDU-M	GYRF-M	AMWI-NS	TUDU-NS	GYRF-NS
AMWI-HA		97.24	92.66									
TUDU-HA	97.24		93.95									
GYRF-HA	92.66	93.95										
AMWI-NA					57.02	56.3						
TUDU-NA				57.02		95.63						
GYRF-NA				56.32	95.63							
AMWI-M								97.45	91.54			

TUDU-M							97.45					
GYRF-M							91.54	91.54				
AMWI-NS											93.91	93.67
TUDU-NS										93.91		
GYRF-NS										93.67	92.84	
